# Proteotoxic stress targeted therapy (PSTT)

**DOI:** 10.18632/oncotarget.284

**Published:** 2011-06-01

**Authors:** Michael Y. Sherman

**Affiliations:** Department of Biochemistry, Boston University School of Medicine

About a decade ago a multiple myeloma patient demonstrated a complete response to a proteasome inhibitor Bortezomib. This case generated a lot of hopes, and after successful clinical trials Bortezomib was approved by FDA and became the drug of choice for this disease. A high sensitivity of multiple myeloma to proteasome inhibition is probably related to extensive production immunoglobulins by the cancer cells. Bortezomib prevents degradation of these proteins, which accumulate and cause a strong ER stress, eventually triggering apoptosis [1, 2]. Unfortunately, many patients show only partial response to Bortezomib, in part due to the development of resistance [3]. Among multiple mechanism of resistance to Bortezomib, cells upregulate expression of molecular chaperones, e.g. Hsp27, which protect from the buildup of abnormal protein species [4-7]. The balance between toxicity of abnormal polypeptides and protective function of chaperones defines the outcome of the treatment with proteasome inhibitors.

Bortezomib has also been tested in trials for treatment of solid tumors, either alone or in combination with other drugs, and results have been promising. The selective sensitivity of various cancer cells to proteasome inhibitors as compared to normal cells is probably because cancer cells have to handle higher levels of abnormal polypeptides, since they are exposed to adverse conditions of tumor microenvironments, oxidative stress and because of genetic instability and associated accumulation of mutant proteins [8]. Accordingly, cancer cells often have increased levels of molecular chaperones, like Hsp72 or Hsp27, as well as the heat shock transcription factor Hsf1 [9]. Furthermore, it was found that cancers have special requirements for these factors, since Hsf1 and hsp72 knockout mice demonstrate dramatic resistance to certain types of cancer, e.g. skin or breast cancer [10-12]. These data led to a concept of the “non-oncogene addiction” of cancers to Hsf1 and molecular chaperones, and predicted that inhibition of chaperones could be a potent novel approach towards development of therapeutics [8]. In fact, there have been number of publications that pharmacological inhibition or siRNA-mediated depletion of Hsf1, Hsp72 or Hsp27 can selectively cause apoptosis or growth inhibition in various types of cancer [9, 13]. Furthermore, anti-sense RNA against Hsp27 has been tested and demonstrated promising results with bladder cancer [14, 15]. Since downregulation of Hsf1 or chaperones increases the generation of abnormal proteins, while inhibition of proteasome blocks their degradation, the combination of these treatments could synergistically precipitate a selective demise of cancer cells. Indeed, it was demonstrated that downregulation or inhibition of Hsf1 can potently enhance sensitivity of cancer cells to Bortezomib [16, 17]. Accordingly, a lot of efforts and resources have been invested in academic labs and industry in development of inhibitors of Hsf1 and various chaperones, but so far there have been no breakthroughs.

The paper by Neznanov et al. [18] offers an alternative approach towards sensitization of cancer cells to proteasome inhibitors. Instead of inhibition of chaperones, this group proposes to cause a buildup of abnormal polypeptides by heat shock. They demonstrated that a mild non-toxic heat shock strongly enhances apoptosis caused by sub-toxic concentrations of Bortezomib. Although there are no data on the effects of heat shock beyond cell culture models, an interesting possibility is that a combination of local hyperthermia and Bortezomib could be beneficial for treatment of localized tumors. Furthermore, since only mild heat treatment was necessary for the synergistic effect with Bortezomib, there is a possibility that the fever-range temperature, which could be triggered by pyrogenes can be sufficient for enhancement of Bortezomib-induced anti-cancer effects. This possibility opens up a range of approaches towards treatment.

As an alternative way of generating abnormal proteins, the authors used puromycin. This inhibitor accepts growing polypeptide chains, and aborts further growth, leading to the release of incomplete chains, which of course cannot fold properly and are toxic. At low sub-toxic concentrations, puromycin could strongly potentiate killing of cancer cells by Bortezomib. In this set of experiments, the authors tested not only cell culture models, but also a mouse model of multiple myeloma. They demonstrated that Bortezomib alone only partially reduced growth of multiple myeloma tumors transplanted into syngenic animals. On the other hand, a combination of Bortezomib and low doses of puromycin led to almost complete inhibition. Overall, the combination of proteasome inhibitors with puromycin, heat shock or other treatments that generate a buildup of abnormal polypeptides represents a novel, and potentially powerful approach towards cancer treatment.
